# *SKA3* is a prognostic biomarker and associated with immune infiltration in bladder cancer

**DOI:** 10.1186/s41065-022-00234-z

**Published:** 2022-05-11

**Authors:** Chenyang Wang, Shasha Liu, Xinhong Zhang, Yan Wang, Peng Guan, Fanyou Bu, Hao Wang, Dawen Wang, Yi Fan, Sichuan Hou, Zhilei Qiu

**Affiliations:** 1 Department of Urology, Qingdao Municipal Hospital, Qingdao University, 266071 Qingdao, Shandong China; 2 Department of Anesthesiology and Surgery, Qingdao Municipal Hospital, Qingdao University, 266071 Qingdao, Shandong China; 3 Department of Traditional Chinese Medicine, Qingdao Municipal Hospital, Qingdao University, 266071 Qingdao, Shandong China; 4 Department of Oncology, Qingdao Municipal Hospital, Qingdao University, 266071 Qingdao, Shandong China

**Keywords:** Spindle and kinetochore-associated complex subunit 3, Bladder cancer, Prognosis, Immune infiltration, Biomarker

## Abstract

**Background:**

Spindle and kinetochore‑associated complex subunit 3 (*SKA3*) has recently been considered a key regulator of carcinogenesis. However, the connection between *SKA3* and immune cell infiltration remains unknown.

**Methods:**

The current study investigated the expression mode, prognostic effect, and functional role of *SKA3* in different tumors, particularly bladder cancer using numerous databases, comprising TIMER, GEPIA, HPA, UALCAN, PrognoScan, and Kaplan–Meier Plotter. Differentially expressed gene and enrichment analyses were implemented on *SKA3* using R packages "edgR" and "clusterProfiler". Immunohistochemistry was further used to validate the expression of *SKA3* gene in bladder cancer. Following that, the relevance of *SKA3* expression to immune infiltration level in bladder cancer was evaluated using TIMER.

**Results:**

Overall, the level of *SKA3* expression in tumor tissue significantly increased than in normal tissue. In bladder cancer and other tumors, patients with high *SKA3* expression levels had worse overall survival (OS) (*p* = *0.016*), disease-specific survival (DSS) (*p* = *0.00004*), and disease-free survival (DFS) (*p* = *0.032*). Additionally, the major molecular functions for *SKA3* included nuclear division, mitotic nuclear division, mitotic sister chromatid segregation, humoral immune response, and cell chemotaxis. Additionally, *SKA3* expression was found to be positively associated with enhanced M2 macrophage and T helper (Th) 2 cell infiltration in bladder cancer.

**Conclusions:**

Our study implies that *SKA3* contributes to M2 macrophage and Th2 cell polarization by acting as an oncogene in bladder cancer. *SKA3* might be a novel biomarker for evaluating prognosis and immune infiltration in bladder cancer.

**Supplementary Information:**

The online version contains supplementary material available at 10.1186/s41065-022-00234-z.

## Introduction

Bladder cancer is the tenth most common carcinoma worldwide, accounting for 3% of all cancer patients [1]. Despite novel treatment strategies, the 5-year survival rate of muscle-invasive bladder cancer does not surpass 70% in the US [2]. Tumor biomarkers are a new kind of investigator that enables clinicians to grasp tumor macroenvironment and microenvironment, allowing for early cancer diagnosis, improved outcomes, and the use of targeted therapy [3, 4]. At present, bladder cancer suffers from a shortage of valid early tumor biomarkers and diagnostic tools. Bladder urothelial carcinoma is deemed an immunogenic malignancy due to its comparatively high tumor mutational load and reactivity to Bacillus Calmette–Guerin treatment and immune checkpoint drugs. As a result, there is a pressing demand for the certification of new immune tumor biomarkers in bladder cancer.

*SKA3* is an element of *SKA* complex, which affects maintaining kinetochore-microtubule interaction in mitosis [5–7]. *SKA3* overexpression has been linked to the occurrence of various cancers. Research exhibited that *SKA3* was somatically mutated in breast cancer, and its ectopic expression promoted tumor progression [8]. *SKA3* overexpression was confirmed in renal cell carcinoma clinical specimens [9]. *SKA3* expression is enhanced in liver cancer tissue compared to normal liver tissue, and inhibiting *SKA3* expression considerably weakens the proliferation of liver cancer cells [10]. Moreover, *SKA3* overexpression causes colorectal adenoma to progress to cancer, whereas knocking out *SKA3* in colorectal adenoma cells greatly reduces cell growth rate, induces G2/M block, and reduces cell migration and invasion [11]. The above findings indicate that *SKA3* develops a significant function in engendering and proliferation of tumors and is a potentially attractive anti-cancer target. However, the role of *SKA3* in bladder cancer remains unknown.

We employed databases such as TIMER, GEPIA, UALCAN, PrognoScan, and Kaplan–Meier plotter for all-around assessment of *SKA3* expression and its correlation with cancer patient prognosis. Additionally, *SKA3* was predicted to be involved in nuclear division, mitotic nuclear division, mitotic sister chromatid segregation, humoral immune response, and cell chemotaxis.The high expression of *SKA3* in bladder cancer was further verified using immunohistochemistry. In addition, we utilized TIMER to investigate the relationship between *SKA3* and immune infiltration in bladder tumor microenvironments. Our findings reveal a potential practical function of *SKA3* in bladder cancer and emphasize a machine-made foundation of *SKA3* influences on M2 macrophage and Th2 cell polarization in tumor microenvironment.

## Materials and methods

### *SKA3* expression analysis in Human Cancers

The expression status of *SKA3* in various cancers was clarified using TIMER (http://cistrome.org/TIMER/) [12]. *SKA3* expression data were obtained and compared in bladder cancer and normal tissue using GEPIA (Gene Expression Profiling Interactive Analysis, http://gepia.cancer-pku.cn/index.html). GEPIA is a dependable web server for tumors and normal gene expression profiles and interactive analyses [13]. To further judge the expression level of *SKA3* in bladder cancer, the protein expression of *SKA3* gene was compared between normal bladder urothelial tissue and bladder urothelial carcinoma tissue using immunohistochemistry provided by HPA database (Human Protein Atlas, https://www.proteinatlas.org/). HPA is a maximum and most detailed database aimed at assessing protein expression state in human tissues and cells, including Tissue Atlas, Pathology Atlas, and Cell Atlas [14]. UALCAN database (http://ualcan.path.uab.edu/) was employed to investigate *SKA3* expression in different molecular subtypes and *TP53* mutations. UALCAN is a user-friendly and interactive web resource for analyzing cancer OMICS data. It is built on PERL-CGI and provides easy access to publicly available TCGA data [15].

### PrognoScan and Kaplan–Meier Plotter database analysis

The correlation between *SKA3* expression and survival in pan-cancer was analyzed in PrognoScan (http://dna00.bio.kyutech.ac.jp/PrognoScan/index.html) and Kaplan–Meier plotter (https://www.kmplot.com/). Specifically, *SKA3* expression level was searched in all available microarray datasets of PrognoScan to determine its relationship with prognosis [16]. The threshold was set as a Cox *P-value* < *0.05*. Kaplan–Meier plotter is a powerful online tool that can evaluate the impact of 54,000 genes on survival in 21 cancer types from TCGA database [17]. We investigated the relevance between *SKA3* expression level and OS and DFS in KIRP, BLCA, OVC, BRCA, LUAD, and COAD (Supplementary Table 1). Hazard ratios (HRs) and log-rank *P*-values were calculated.

### DEG and enrichment analyses

Using "limma" package in R software (version 4.1.1, www.r-project.org), we divided TCGA-BLCA samples into quartiles based on *SKA3* expression [18]. The group with the lowest 25% of *SKA3* expression was defined as the low expression group, and the group with the highest 25% was defined as the high expression group. Then, "edgeR" package was used to screen the significant differentially expressed genes (DEGs) in low and high expression groups of *SKA3* based on | log2fc |> 1 and Benjamin Hochberg adjusted *P* < *0.01*. Gene ontology (GO) analysis was executed via EnrichGO effect in R package "clusterProfiler". Kyoto encyclopedia of genes and genomes (KEGG) analysis was executed via EnrichKEGG effect in R package "clusterProfiler".

### Immunohistochemistry

Twenty-five bladder cancer tissues and 17 chronic inflammatory tissues of the bladder mucosa collected from the Affiliated Qingdao Municipal Hospital of Qingdao University were subjected to immunohistochemistry staining to detect the expression of *SKA3*. Immunostaining was evaluated by two pathologists from the pathology department. Staining for each of the proteins was scored using the methods of modified immunoreactive score (IRS). The intensity of staining was scored as 0 negative/weak; 1 moderate; 2 strong. The percentage of positive expression was categorized as: 1 (< 25%); 2 (25–50%); 3 (50–75%); 4 (75–100%). The scores of staining intensity and staining range were subsequently multiplied to generate IRS. Finally, the IRS of bladder cancer tissues and chronic inflammatory tissues of the baldderl mucosa was compared using an independent sample t-test using SPSS 26.0. The threshold for statistical significance was P < 0:05. The Ethics Committee of the Affiliated Qingdao Municipal Hospital of Qingdao University approved the above experiments.

### Immune cell infiltration

The correlation between *SKA3* expression level and immune infiltration was verified using TIMER database [12]. TIMER is a classical and authoritative database, which utilizes a deconvolution beforehand established statistical means and analyzes and visualizes the abundance of immune cell infiltration in a specific tumor type [19]. We researched the connection between *SKA3* expression and immune infiltrates level, containing B cells, CD4^+^ T cells, CD8^+^ T cells, macrophages, neutrophils, and dendritic cells. In addition to the general analysis of immune infiltration, we gathered about 40 distinct immune checkpoint genes and investigated the relationship between *SKA3* expression level and immune gene expression in pan-cancer. Furthermore, we investigated the link between *SKA3* expression level and a few immune cell markers. These gene markers include markers of Th2 cells and M2 macrophages [20]. Scatterplots were employed to reflect the correlation between *SKA3* and each immune gene marker.

### Data collection and statistical analysis

RNA sequencing data, gene expression data, and corresponding clinical information of TIMER, GEPIA, UALCAN, and Kaplan–Meier plotter databases were provided by TCGA database, and Prognoscan data were provided by GEO database. The inclusion and exclusion criteria of patients were implemented using TCGA and GEO databases. Survival curves were generated using PrognoScan and Kaplan–Meier plots. The results of Kaplan–Meier plots, PrognoScan, and GEPIA are displayed with HR and P or Cox *P*-values from a log-rank test. In Kaplan–Meier plots, patients were divided into high and low expression groups according to the median. In PrognoScan, patients were divided into high and low expression groups according to the best cutoff, which is derived by computing all potential cutoff values between lower and higher quartiles and using the best performing threshold as a cutoff. Gene expression data (FPKM normalized) for bladder cancer tissue samples in the Cancer Genome Atlas (TCGA) were downloaded from Genome Data Commons (https:// portal.gdc.cancer.gov). For TCGA dataset, RNA sequencing data (FPKM values) were transformed into transcripts per kilobase million (TPM) values and then normalized using Z-score method. *P*-values < 0.05 were considered statistically significant.

## Results

### High *SKA3* expression in bladder cancer

**Fig. 1:**
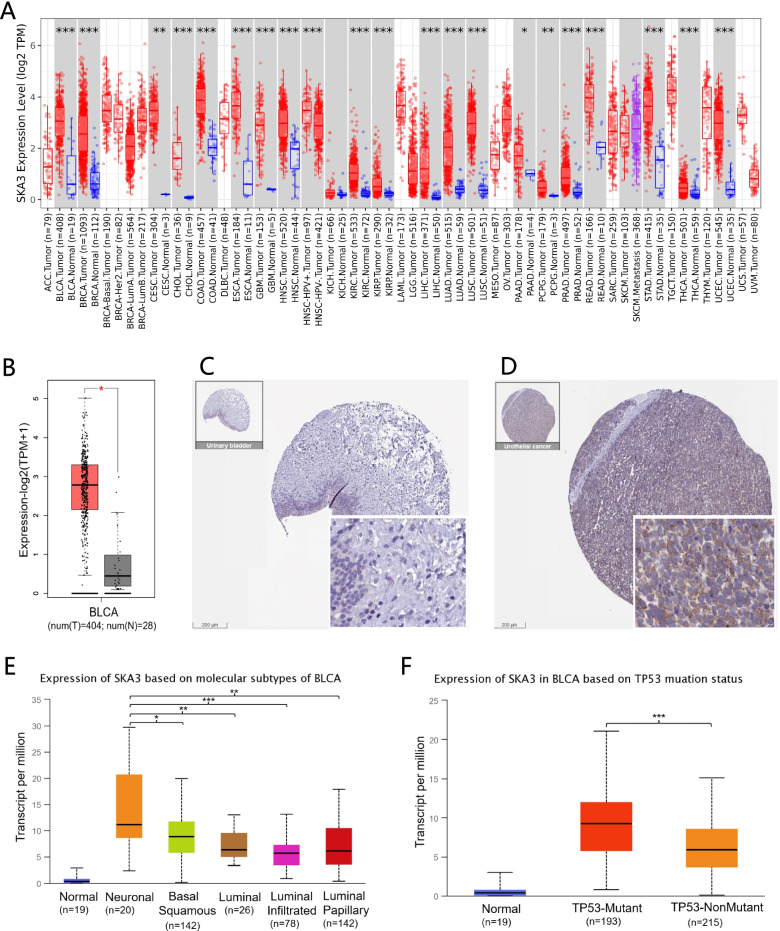
Pan-cancer *SKA3* expression analysis. **A** *SKA3* expression level in tumor and normal tissues in TCGA pan-cancer data using TIMER. **B** The comparison of RNA-seq expression levels between bladder cancer tissue (*n* = 404) and normal control tissue (*n* = 28), *indicates a significant difference (*p* < 0.05). **C** *SKA3* protein level in normal urinary bladder tissues (quantity: none; intensity: negative; staining: not detected; antibody HPA03972). **D** *SKA3* protein level in bladder urothelial carcinoma (quantity: 75%-25%; intensity: weak; staining: low; antibody: HPA03972). **E** *SKA3* expression based on molecular subtypes on BLCA. **F** *SKA3* expression based on TP53 mutation status of BLCA. *P < 0.05, **P < 0.01, ***P < 0.001

To investigate the distribution of *SKA3* expression in different tumors, *SKA3* expression in each cancer type is compared to that of normal tissue samples (reference control) in TIMER database. The discrepancy in *SKA3* expression levels between tumors and corresponding normal tissues is depicted in Fig. 1A. *SKA3* levels were obviously higher in BLCA, BRCA, CESC, CHOL, COAD, ESCA, GBM, HNSC, KIRC, KIRP, LIHC, LUAD, LUSC, PAAD, PCPG, PRAD, READ, STAD, THCA, and UCEC (Supplementary Table 1) than in their respective normal tissues. Interestingly, none of tumors expressed at a lower level than their normal tissues counterparts.

In addition, RNA-seq of *SKA3* from TCGA bladder cancer dataset and GTEx dataset revealed that *SKA3* expression was obviously higher in bladder cancer tissue (*n* = 404) than in normal bladder samples (*n* = 28) (Fig. 1B). Additionally, we confirmed *SKA3* expression in bladder cancer using immunohistochemical staining in HPA database. *SKA3* protein expression was enhanced in bladder urothelial carcinoma tissue than normal bladder urothelial tissue in Figs. 1C and D. In contrast to normal bladder tissue, the results confirmed that *SKA3* was overexpressed in bladder cancer tissues. We further analyzed *SKA3* expression in various molecular subtypes and *TP53* mutation using TCGA bladder cancer dataset. The results proclaimed that *SKA3* expression was much higher in neuronal type than in other four subtypes, and *SKA3* expression was also higher in *TP53* mutant type than in *TP53* nonmutant type (Figs. 1E and F).

### Correlation between cancer patient prognosis and *SKA3* expression level

**Fig. 2:**
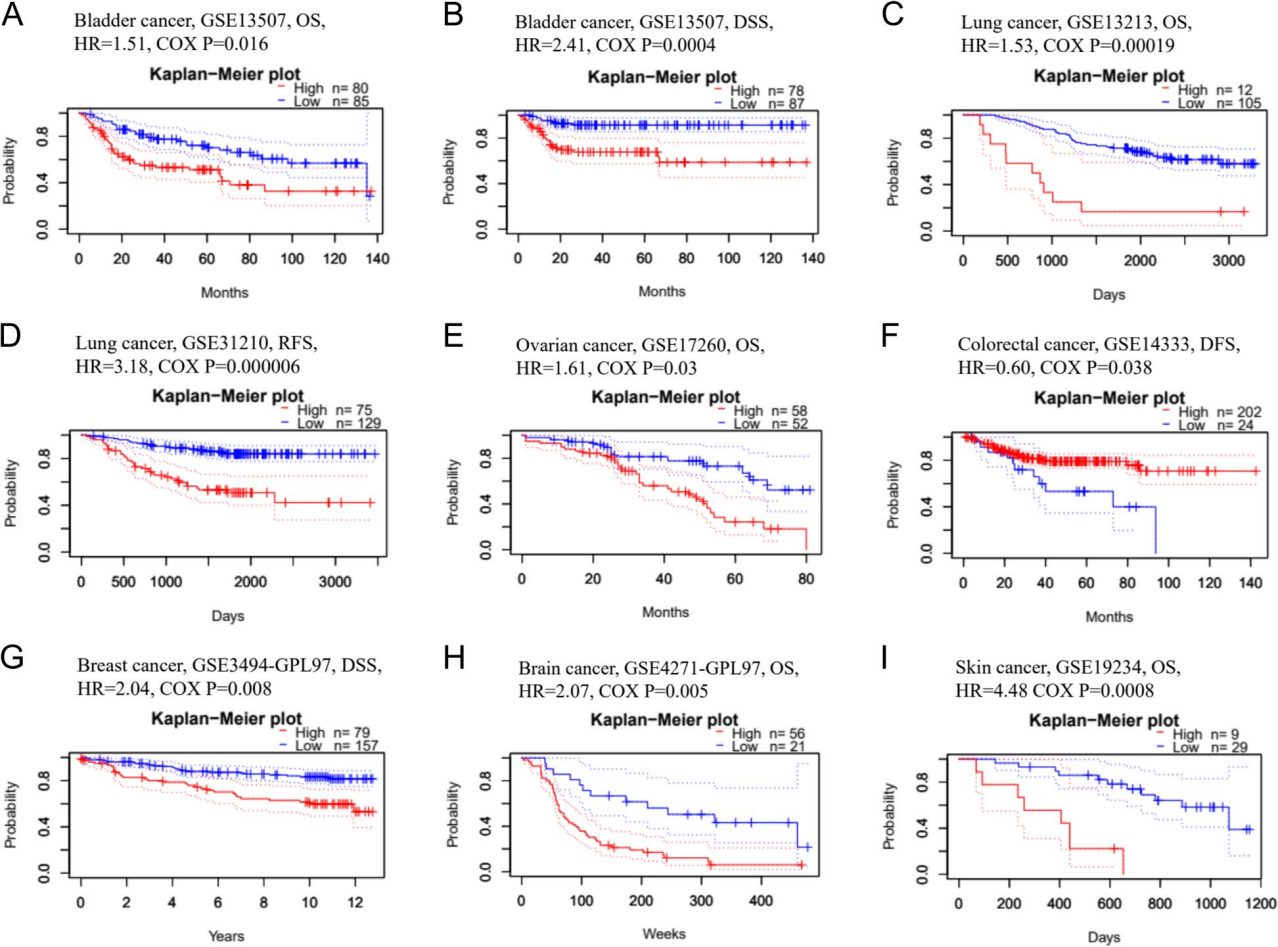
Kaplan Meier survival curve assessed the relationship between *SKA3* expression level and prognosis in Prognoscan. **A**, **B** OS (overall survival) and DSS (disease-specific survival) in cohort GSE13507 of bladder cancer. **C** OS in cohort GSE13213 of lung cancer. **D** RFS (relapse-free survival) in cohort GSE31210 of lung cancer. **E** OS in cohort GSE31210 of ovarian cancer. **F** DFS (disease-free survival) in cohort GSE14333 of colorectal cancer. **G** DSS in cohort GSE3499-GPL97 of breast cancer. **H** OS in cohort GSE4271-GPL97 of brain cancer cohort. **I** OS in cohort GSE19234 of skin cancer

Then, we explored the prognostic value of *SKA3* in various kinds of carcinomas using two powerful databases. In PrognoScan, we investigated the association between *SKA3* expression level and the prognosis of multiple cancers. Notably, *SKA3* expression was significantly associated with seven kinds of cancers, including bladder, lung, ovarian, colorectal, breast, brain, and skin cancers (Fig. 2). In particular, *SKA3* developed a pernicious effect on six kinds of these cancers, including bladder (OS: *Cox P* = *0.016*, HR = 1.51, gross number = 165; DSS: *Cox P* = *0.00004*, HR = 2.41, gross number = 165), lung [OS: *Cox P* = *0.00019*, HR = 1.53, gross number = 117; RFS (relapse-free survival): *Cox P* = *0.000006*, HR = 3.18, gross number = 204], ovarian (OS: *Cox P* = *0.03*, HR = 1.61, gross number = 110), breast (DSS: *Cox P* = *0.0087*, HR = 2.04, gross number = 236),brain(OS: *Cox P* = *0.005*, HR = 2.07, gross number = 77), and skin cancers (OS: *Cox P* = *0.00084*, HR = 4.48, gross number = 38). Meanwhile, *SKA3* developed a conservatory effect in colorectal cancer (DFS: *Cox P* = *0.0385*, HR = 0.60, gross number = 226).

**Fig. 3:**
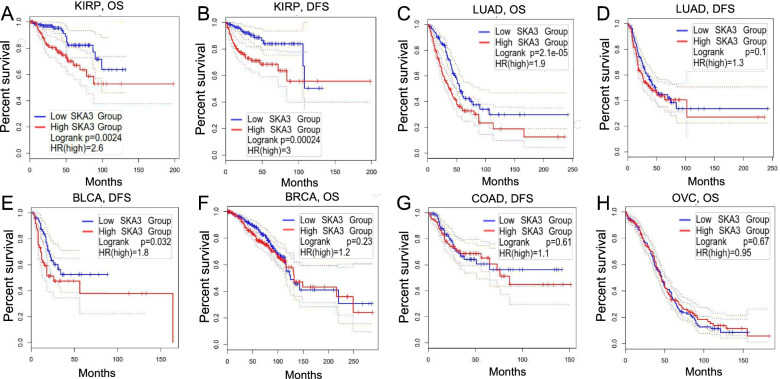
Kaplan–Meier survival curves evaluated the connection between *SKA3* expression and prognosis in various cancer kinds using Kaplan–Meier plotter. **A**, **B** OS and DFS survival curves of KIRP. **A**, **B** OS and DFS survival curves of LUAD. **E** DFS survival curves of BLCA. **F** OS survival curves of BRCA. **G** DFS survival curves of COAD. **H** OS survival curves of OVC

Meanwhile, Kaplan–Meier curve analysis was executed to investigate *SKA3*-related survival using Kaplan–Meier plotter and the Cancer Genome Atlas (TCGA) datasets because PrognoScan information is primarily extracted from Gene Expression Omnibus (GEO) database. Notably, we unexpectedly found *SKA3* as a pernicious prognostic element in KIRP (OS: HR = 2.6, log-rank *P* = *0.0024*; DFS, HR = 3, log-rank *P* = *0.00024*) (Figs. 3A and B). For LUAD, *SKA3* was identified to play a detrimental factor (OS: HR = 1.9, log-rank *P* = *2.1e-05*). However, *SKA3* had no obvious significance in LUAD (DFS: HR = 1.3, log-rank *P* = *0.1*) (Figs. 3C and D). The findings for breast, colorectal, and ovarian cancers were inconsistent with those on PrognoScan, as high *SKA3* expression levels had no discernible effect on the prognosis of three cancers, including BRCA (OS: HR = 1.2, log-rank *P* = *0.23*) (Fig. 3F), COAD (DFS: HR = 1.1, log-rank *P* = *0.61*) (Fig. 3G), and OVC (OS: HR = 0.95, log-rank *P* = *0.67*) (Fig. 3H). Specifically, only BLCA (DFS: HR = 1.8, log-rank *P* = *0.032*) (Fig. 3E) results were consistent with previous predictions using PrognoScan. Consequently, high *SKA3* expression is an independent risk factor for bladder cancer patients, rather than a protective factor.

### Gene ontology and KEGG enrichment analysis

**Fig. 4:**
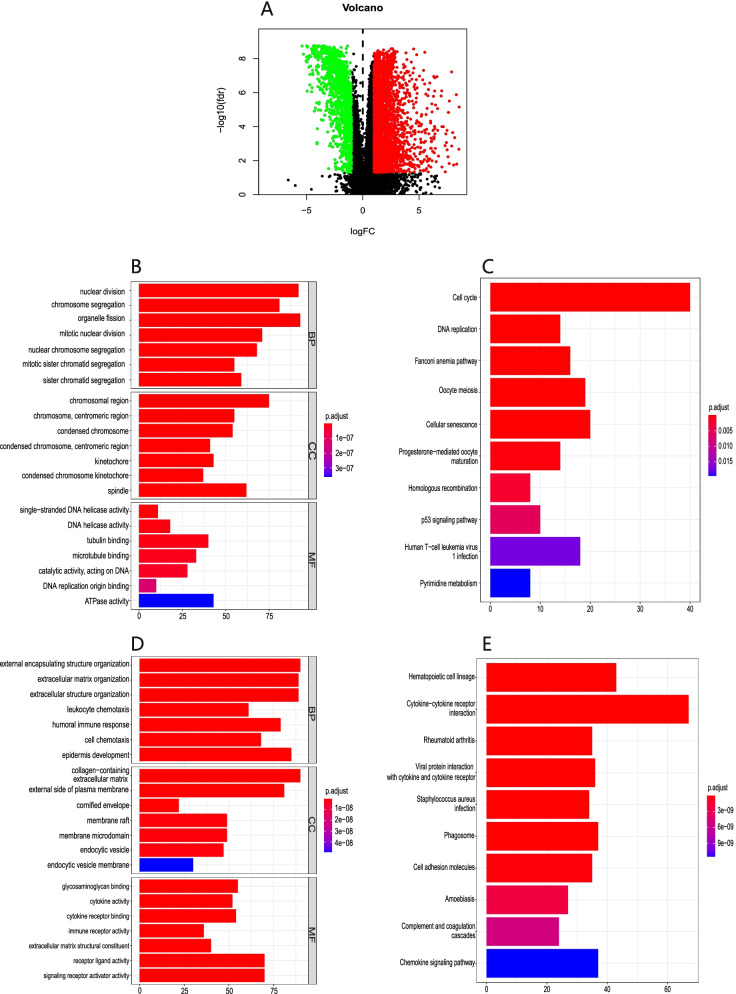
Function and pathway enrichment analyses for *SKA3* in bladder cancer. **A** A volcano plot of all DEGs from indicated TCGA microarray data. Green dots represent significantly downregulated genes, and red dots represent significantly upregulated genes. **B** Gene ontology analysis of upregulated group, containing biological processes (BP), molecular function (MF), and cell component (CC). **C** Kyoto encyclopedia of genes and genomes analyzes pathways of upregulated group. **D** Gene ontology analysis of downregulated group. **E** Kyoto encyclopedia of genes and genomes analyzes pathways of downregulated group

To predict *SKA3* function, TCGA-BLCA samples were divided into two groups according to their *SKA3* expression quartile. Differentially expressed gene analysis was performed to determine 847 differentially expressed genes (DEGs) (|log2FC|> 1, Benjamin Hochberg adjusted *P* < *0.01*). All genes were also displayed in a volcano plot to illustrate their distribution (Fig. 4A). To explore the latent functions associated with 847 genes, they were divided into upregulated group and downregulated group, and the GO enrichment analysis was conducted using biological process, molecular function, and cellular component in bubble plots (Fig. 4B and D). The results revealed that *SKA3* was primarily linked to, nuclear division,organelle fission,mitotic nuclear division,mitotic sister chromatid segregation in upregulated group; and linked to, extracellular matrix organization, extracellular structure organization, humoral immune response,cell chemotaxis in downregulated group. Following that, KEGG pathway analysis revealed that the upregulated group was enriched and interacted with in cell cycle,DNA replication,oocyte meiosis,and p53 signaling pathway (Fig. 4C); and the downregulated group was enriched and interacted with in Cytokine − cytokine receptor interaction, Viral protein interaction with cytokine and cytokine receptor, Cell adhesion molecules, and Chemokine signaling pathway (Fig. 4E). These studies confirm that *SKA3* is linked to a few malignancy-related pathways that lead to bladder cancer.

### Confirmation of *SKA3* Expression by Immunohistochemistry

**Fig. 5:**
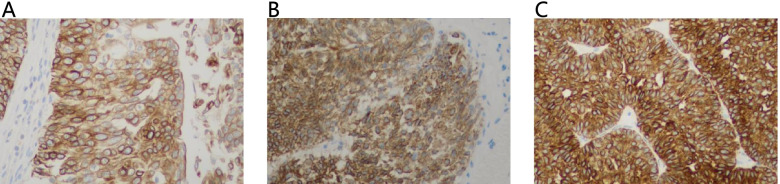
Results of *SKA3* immunohistochemistry. **A** The weak positive expression of *SKA3* in bladder cancer tissues. **B** The moderate strong positive expression of *SKA3* in bladder cancer tissues. **C** The strong positive expression of *SKA3* in bladder cancer tissues

To investigate the expression of *SKA3* in baldder cancer, *SKA3* protein levels were examined using immunohistochemistry in 25 cases of bladder cancer tissues and 17 cases of normal bladder tissues. We found negative *SKA3* immunostaining in normal urothelial tissues. Increased cytoplasm *SKA3* staining was found in 21 of 25 cases of bladder cancer (Fig. 5). An independent sample t-test of SPSS 26.0 was used to compare the IRS between the two groups, and the results indicated that the protein expression of *SKA3* in bladder cancer tissues was significantly higher than that in noncancerous tissues (*P* < 0.05).

### Correlation between immune cell infiltration and *SKA3*

**Fig. 6:**
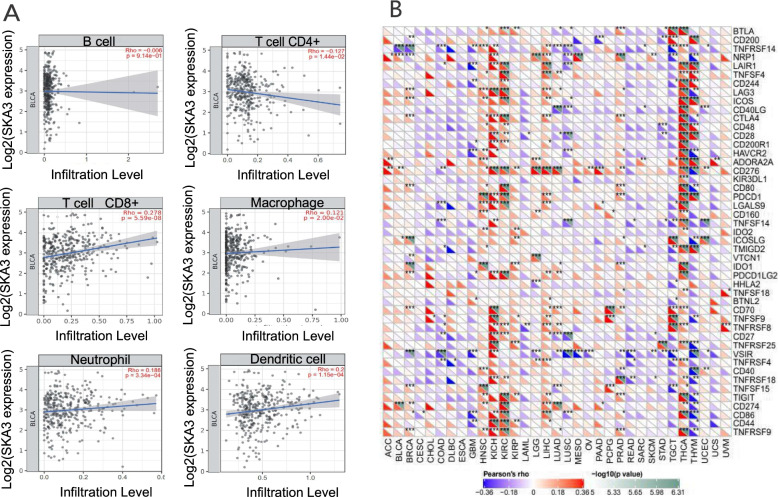
Correlation between *SKA3* expression level with immune infiltration. **A** Correlation between *SKA3* expression level and infiltration level of each immune cell in bladder cancer in TIMER. **B** Correlation between *SKA3* and immune checkpoint genes are shown in a heatmap. *P < 0.05, **P < 0.01, ***P < 0.001

Immune cell infiltration surrounding tumors was confirmed to be intimately associated with the clinical outcome of cancer patients [21, 22]. Consequently, we defined if *SKA3* expression was connected to immune infiltration level by examining their correlation in bladder cancer using TIMER. This analysis revealed that *SKA3* expression level in BLCA was positively correlated with the infiltration levels of CD8 + T cells, macrophages, neutrophils, and dendritic cells. Besides, it had a negative association with CD4^+^ T cell infiltration levels (Fig. 6A). The preceding investigation indicated that *SKA3* influences patient prognosis via its interaction with immune cell infiltration in bladder cancer. We then investigated the connection between *SKA3* and immune checkpoint genes in TIMER database. *SKA3* was closely correlated with immune checkpoint genes in most tumors, including bladder cancer (Fig. 6B).

### *SKA3* expression was related to M2 macrophage and Th2 cell infiltration and polarization

**Fig. 7:**
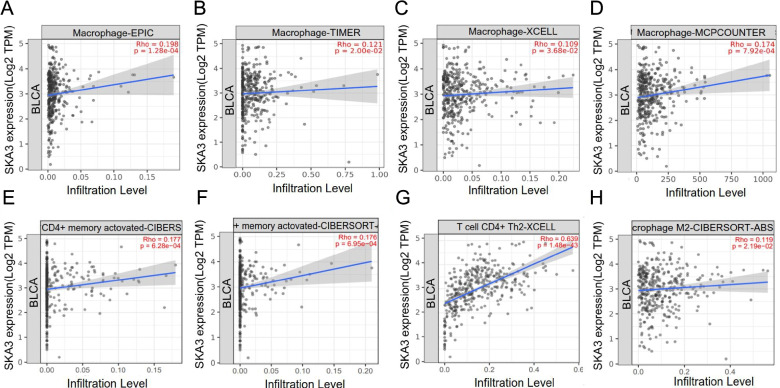
Connection among *SKA3* expression with macrophage and CD4 + T cell infiltration and polarization. **A**–**D** Correlation between *SKA3* expression and macrophage infiltration levels using four different algorithms: EPIC, TIMER, XCELL, and MCPCOUNTER. **E**, **F** Correlation between *SKA3* expression and CD4 + memory activated T cells infiltration levels using two different algorithms: CIBERSOFT and CIBERSOFT-ABS. **G** Correlation between *SKA3* expression and CD4 + Th2 cell infiltration levels using algorithms of XCELL. **H** Correlation between *SKA3* expression and M2 macrophage infiltration levels using CIBERSOFT-ABS algorithms

**Fig. 8:**
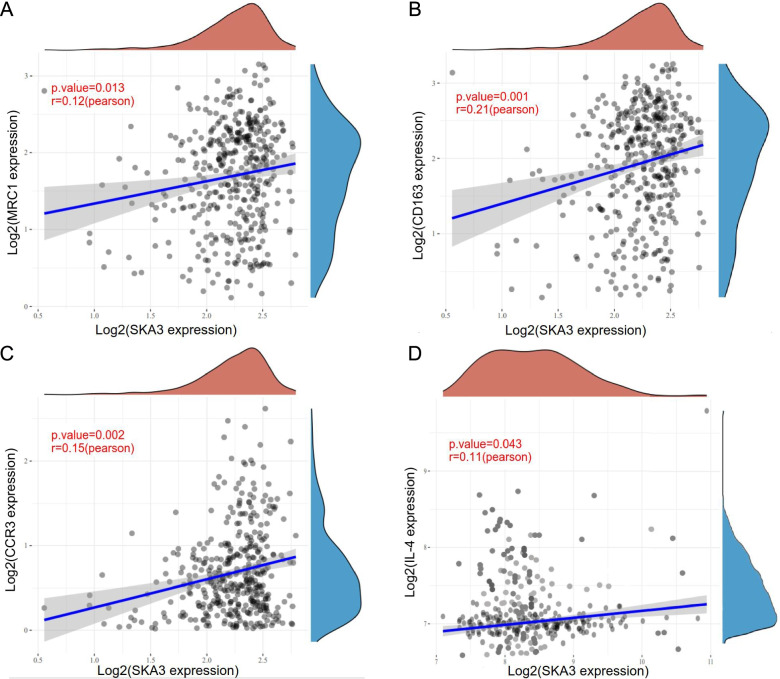
Correlation between *SKA3* expression and gene markers of M2 macrophages and TH2 cells. **A** Correlation among *SKA3* expression with MRC1 in bladder cancer. **B** Correlation among *SKA3* expression with CD163 in bladder cancer. **C** Correlation among *SKA3* expression with CCR3 in bladder cancer. **D** Correlation among *SKA3* expression with IL-4 in bladder cancer

The immune infiltration state in tumor microenvironment (TME) can affect patient prognosis, and previous results indicate that *SKA3* overexpression was connected to deleterious prognosis in bladder cancer patients. This implies that prominent *SKA3* expression promotes the proliferation and metastasis of bladder tumors, which may be tightly associated with immunosuppressive tumors. Accordingly, we further explored whether *SKA3* expression was linked to immune infiltration levels of macrophages and CD4^+^ T cells. *SKA3* expression was found to be positively associated with macrophage infiltration level using four algorithms, including EPIC (*R* = 0.198, *P* = *1.28E-04*) (Fig. 7A), TIMER (*R* = 0.121, *P* = *2.00E-02*) (Fig. 7B), XCELL (*R* = 0.109, *P* = *3.68E-02*) (Fig. 7C), and MCPCOUNTER (*R* = 0.174, *P* = *7.92E-04*) (Fig. 7D). Besides, *SKA3* expression was positively linked to CD4 + memory-activated T cells infiltration level in two different algorithms, including CIBERSOFT (*R* = 0.177, *P* = *6.28E-04*) (Fig. 7E) and CIBERSOFT-ABS (*R* = 0.176, *P* = *6.95E-04*) (Fig. 7F). For CD4^+^ T cell polarization, *SKA3* expression was positively linked to Th2 cells polarization (*R* = 0.639, *P* = *1.48E-43*) (Fig. 7G). For macrophage polarization, *SKA3* expression was positively correlated with M2 polarization (*R* = 0.119, *P* = *2.19E-02*) (Fig. 7H). In addition, *SKA3* expression in bladder cancer was significantly correlated with M2 macrophage markers, including *MRC1* (Fig. 8A) and *CD163* (Fig. 8B), and Th2 cell markers, including *CCR3* (Fig. 8C) and *IL-4* (Fig. 8D). The above findings support that high *SKA3* expression is connected with immunosuppressive microenvironment of bladder cancer via infiltration and polarization of M2 macrophages and Th2 cells. M2 macrophages are derived from M1 activation by factors such as *IL-4* and *IL-13* and have the potential to suppress immune responses, promote angiogenesis, tissue repair and promote tumor growth. Tumor tissues mostly secrete Th2-like cytokines and the body is in a state of Th2 cell dominance as one of the mechanisms of tumor immune escape.

## Discussion

*SKA3* is required to maintain spindle checkpoint silencing and mitotic chromosomal cohesion and ensure timely mitosis [23–25]. Although *SKA3* has been linked to the occurrence and development of numerous diseases, it has not been widely researched in various tumors. As a result, *SKA3* impact on cancer prognosis, progression, and treatment must be identified and proven urgently. Previous studies reported that *SKA3* could promote PI3K/AKT pathway to accelerate cell proliferation and migration in cervical cancer [26]. Meanwhile, *SKA3* expression was higher in glioblastoma than in normal tissues, and it was verified that patients with high *SKA3* expression have a shorter survival time [27]. A recent study reported that high *SKA3* expression is associated with poor clinical outcomes and muscle-invasive bladder cancer progression, and promotes bladder cancer cell proliferation by accelerating G2/M transition [28].

Based on our results, we employed TCGA data in TIMER to investigate *SKA3* expression and its prognostic impact on various cancer types, demonstrating that *SKA3* was highly expressed in 20 different tumors compared to normal tissues. In addition, in GEPIA and HPA databases, *SKA3* was highly expressed in bladder cancer relative to normal bladder tissue. Additionally, using Prognoscan database, we affirmed that high *SKA3* expression in tumor tissues is significantly connected with poor prognosis of patients with bladder, lung, ovarian, breast, brain, and skin cancers. On the contrary, high *SKA3* expression in colorectal cancer was associated with favorable survival. Meanwhile, Kaplan–Meier Plotter analysis revealed that high *SKA3* expression was correlated with inferior OS and DFS in KIRP and BLCA but had no significant impact on OS and DFS in BRCA, COAD, and OVC. In different databases, prognostic disparities in *SKA3* expression levels of various cancers result from the heterogeneity of material gathering methods and the latent mechanisms underlying distinct biological characteristics. Whereas, based on research results in different databases, we determined the accordant prognostic function of *SKA3* on BLCA. This finding is consistent with previous studies [28]. To investigate the expression of *SKA3* in baldder cancer, *SKA3* protein levels were examined using immunohistochemistry in 25 cases of bladder cancer tissues and 17 cases of normal bladder tissues. Increased cytoplasm *SKA3* staining was found in 21 of 25 cases of bladder cancer, and the results indicated that the protein expression of *SKA3* in bladder cancer tissues was significantly higher than that in noncancerous tissues. It is reported that molecular subtypes are associated with prognosis and immune status of bladder cancer. Based on data from 412 cases of bladder cancer RNA sequencing, TCGA research team proposed five molecular subtypes: luminal-papillary subtype, luminal-infiltrated subtype, luminal subtype, basal-squamous subtype, and neuronal subtype. The neuronal subtype showed high levels of *TP53* and *RB1* mutations and had the worst survival of mRNA expression subtypes [29]. Our investigation revealed that *SKA3* expression in neuronal subtype was much higher than that in other four subtypes, and *SKA3* expression was also higher in *TP53* mutant type than in *TP53* nonmutant type. Overall, the above research explicitly illustrates that *SKA3* can be a latent biomarker to forecast prognosis and molecular subtypes of tumors.

Numerous bladder cancer tumor biomarkers have been identified and validated to date [30, 31]. However, with rapid development of bladder cancer immunotherapy, the discovery of tumor biomarkers or immune checkpoints pertaining to tumor immune microenvironment is critical. However, the correlation between *SKA3* and immune infiltration of bladder cancer has not been studied. Another significant aspect of our research indicates that *SKA3* expression is associated with various levels of immune infiltration in bladder cancer. TME is a distinct metabolic niche, which differs according to the kind of tumor and influenced organs [32, 33]. TME is produced due to well-orchestrated reciprocal interactions between cancer cells and surrounding stromal and immune cells [34]. Immune cells in tumor microenvironment are a key factor of tumor tissues. Our results illustrated that *SKA3* expression correlates positively with the infiltration level of CD8^+^T cells, macrophages, neutrophils, and dendritic cells in BLCA. Meanwhile, significant relationships between *SKA3* and immune checkpoint gene expression in pan-cancer indicate that *SKA3* plays a key function in regulating tumor immunology. Tumor cells acquire strategies to evade immune surveillance during the prolonged interaction between cancer and the immune system. Tumors may abscond immunity via drawing immune suppressive cells, such as tumor-associated macrophages (TAMs). TAMs usually possess M2 phenotypes, suppress adaptive immunity, and facilitate TME that can accelerate cancer progression. Several researches have confirmed that M2 macrophages with a higher infiltration level are positively correlated with poor histopathological characteristics, including higher tumor and lymph node stage and histological grade in bladder cancer patients [35–37]. GO results revealed that *SKA3* was strongly associated with nuclear division, mitotic nuclear division, mitotic sister chromatid segregation, humoral immune response, and cell chemotaxis. Meanwhile, KEGG analysis indicated that *SKA3* was involved in the cell cycle, DNA replication, p53 signaling pathway, Cytokine − cytokine receptor interaction, and Chemokine signaling pathway. By analyzing the relationship between *SKA3* and macrophage infiltration in BLCA, we discovered that macrophages in four different algorithms and M2 macrophages had significantly positive correlations with *SKA3* expression levels. Moreover, *SKA3* expression was positively correlated with gene markers of M2 macrophage such as *MRC1* and *CD163* in bladder cancer.

As immunotherapy becomes a valid treatment method for cancer, CD4^+^ T helper cells have received extensive attention as a crucial component of immune response [38]. Meanwhile, activated CD4^+^ T cells can differentiate into various T helper cells with different effector functions based on their phenotypes, cytokine profiles, and functionality, such as Th1, Th2, Th17, follicular helper T, and regulatory T cells [39–41]. Tumor cells and specific immune cells that differentiate towards Th2 phenotype are the best correlative immune escape mechanisms. For Th2 differentiation, *IL-4* is essential for launching and sustaining Th2 phenotypes [34]. By combining with *IL-4* receptors, *IL-4*, a typical Th2 cytokine, induces naive CD4^+^ T cells to develop into Th2 cells [42]. Following differentiation, TH2 cells secrete *IL-4*, *IL-5*, *IL-10*, *IL-13*, and *IL-17*. Additionally, *IL-4*, *IL-5*, and *IL-13* have been demonstrated to accelerate tumor proliferation and metastasis [43, 44]. Our findings indicated that infiltration levels of activated CD4^+^ memory T cells and Th2 cells were highly linked to *SKA3* expression in BLCA. Concurrently, *SKA3* expression was positively correlated with the expression level of Th2 cell markers (*IL-4* and *CCR3*) in bladder cancer.

In conclusion, *SKA3* can influence the prognosis of bladder cancer patients and is associated with immune infiltration. In particular, *SKA3* may promote bladder cancer growth and metastasis by influencing the differentiation of M2 macrophage and Th2 cells. Therefore, *SKA3* can represent a valuable prognostic biomarker for bladder cancer.

## Supplementary Information

**Additional file 1.**

## Data Availability

The datasets supporting the conclusions of this article are included within the article. Materials are available from the corresponding author on reasonable request.

## Abbreviations

DSS: Disease-specific survival
DFS: Disease-free survival
GEPIA: Gene Expression Profiling Interactive Analysis
GO: Gene Ontology
GEO: Gene Expression Omnibus
HPA: Human Protein Atlas
HRs: Hazard ratios
KEGG: Kyoto Encyclopedia of Genes and Genomes
OS: Overall survival
RFS: Relapse-free survival
*SKA3*: Spindle and kinetochore‑associated complex subunit 3
Th: T helper
TCGA: The Cancer Genome Atlas
TME: Tumor microenvironment
TAMs: Tumor-associated macrophages
